# Loss of the DCHS1 Intracellular Domain Expands Neurogenic Proliferation and Generates Van Maldergem-like Neurodevelopmental Defects

**DOI:** 10.3390/cells15070587

**Published:** 2026-03-26

**Authors:** Kathryn Byerly, Cayla Wolfe, Magdalena Brei, Hannah Parris, Savannah Fisher, Aimee Alston, Hanmei Dong, Peng Chen, Hai Yao, Fulei Tang, Jan Guz, Sarah Dooley, Taylor Nelson, Brian Loizzi, Ranan Phookan, Cortney Gensemer, Sunil Patel, Russell A. Norris

**Affiliations:** 1 Department of Regenerative Medicine and Cell Biology, Medical University of South Carolina, Charleston, SC 29407, USA; byerlyk@musc.edu (K.B.); wolfecay@musc.edu (C.W.); brei@musc.edu (M.B.); parrihan@musc.edu (H.P.); fishesav@musc.edu (S.F.); alstonae@g.cofc.edu (A.A.); dooleys@musc.edu (S.D.); petrucct@musc.edu (T.N.); loizzi@musc.edu (B.L.); phookan@musc.edu (R.P.); gensemer@musc.edu (C.G.); 2 Clemson-MUSC Bioengineering Program, Department of Bioengineering, Clemson University, Charleston, SC 29425, USA; dongha@musc.edu (H.D.); chenpe@musc.edu (P.C.); yaoh@musc.edu (H.Y.); 3 Department of Oral Health Sciences, Medical University of South Carolina, Charleston, SC 29425, USA; 4 Department of Comparative Medicine, Medical University of South Carolina, Charleston, SC 29407, USA; fulei@musc.edu (F.T.); guzj@musc.edu (J.G.); 5 Department of Neurosurgery, Medical University of South Carolina, Charleston, SC 29407, USA; patels@musc.edu

**Keywords:** Dachsous, Hippo signaling, neurodevelopment, heterotopia, cell polarity

## Abstract

Van Maldergem Syndrome (VMS) is a rare autosomal recessive disorder caused by pathogenic variants in the atypical cadherin genes *DCHS1* or *FAT4* and is marked by craniofacial, skeletal, and neurodevelopmental abnormalities. Although DCHS1–FAT4 binding is mediated by their respective extracellular domains, the in vivo function of the DCHS1 intracellular domain (ICD) is poorly defined. To test its function, we generated mice in which the DCHS1 ICD was deleted and replaced with a V5 epitope tag (*Dchs1*^Δ*ICD-V5*^). Homozygous *Dchs1*^Δ*ICD-V5/*Δ*ICD-V5*^ mice are viable but exhibit VMS-like craniofacial flattening with enlarged fontanelles and reduced palatine/maxillary structures, along with airway cartilage abnormalities including reduced mineralization and decreased tracheal circularity. In periventricular regions, wild-type DCHS1 expression shows polarized localization, whereas mice with the ICD deletion exhibit altered cell polarization within the subventricular zone, concomitant with changes in neural cellular distribution. Neonatal brains display reduced pYAP1: YAP1 ratios and increased Ki67+ proliferation with greater Ki67–neuronal co-localization within the periventricular zone. Together, these data identify the DCHS1 ICD as a critical effector for DCHS1 signaling and a regulator of polarity-dependent growth, with associated changes in Hippo pathway activity during craniofacial and neural morphogenesis. Additionally, our data establish *Dchs1*^Δ*ICD-V5/*Δ*ICD-V5*^ mice as a model that recapitulates core features of VMS, thereby allowing new mechanistic discoveries into its pathogenesis.

## 1. Introduction

**Figure 1:**
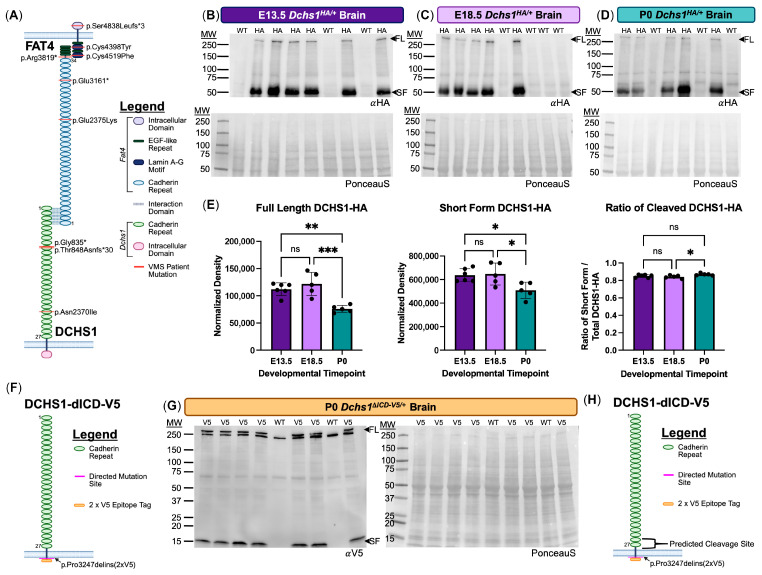
DCHS1 protein cleavage in developing mouse brain. (**A**) DCHS1 and FAT4 variants identified in Van Maldergem Syndrome patients and their position within specific domains of each cadherin protein. (**B**–**D**) Western blot analyses of brain lysate from individual mice in a litter of *Dchs1^HA^*^/+^ and *Dchs1*^+/+^ pups at E13.5, E18.5, and P0 showing the presence of full-length (FL) and a short-form (SF) fragment following blotting with an HA-specific antibody. Ponceau-S was used as a protein loading control. (**E**) Quantification of normalized density from FL, SF, and their ratio at E13.5 (*n* = 6), E18.5 (*n* = 5), and P0 (*n* = 5) in B-D. (**F**) Protein map of DCHS1-dICD-V5 protein showing replacement of the ICD with a 2X V5 epitope tag. (**G**) Whole-brain lysates from P0 *Dchs1*^ΔICD-V5^ mice showing presence of the full length (FL) and a short form (SF). Ponceau-S was used as a protein loading control. (**H**) Bracket representing potential region that contains DCHS1 cleavage site (C.S.). ns = not significant, * *p* < 0.05; ** *p* < 0.01; *** *p* < 0.001.

Craniofacial and neural morphogenesis is a complex developmental process that requires coordinated cell polarity, proliferation, and differentiation across multiple tissue layers [1]. The interruption of these processes underlies a spectrum of congenital anomalies, including Van Maldergem Syndrome (VMS). VMS is a rare, autosomal recessive disorder characterized by craniofacial dysmorphology, periventricular neuronal heterotopia, and skeletal defects [2,3,4,5]. Pathogenic variants in the *DCHS1* or *FAT4* genes have been identified as the primary genetic causes of VMS [2] (Figure 1A), underscoring the critical roles of DCHS1–FAT4 signaling in vertebrate tissue organization and morphogenesis.

DCHS1 and FAT4 are large atypical cadherins that function respectively as a ligand–receptor pair to mediate planar cell polarity (PCP) [1] and regulate the Hippo signaling pathway [6]. In the developing brain and neural tube, DCHS1–FAT4 interactions use these pathways to ensure proper neuronal migration and cortical layering [3]. The loss of either DCHS1 or FAT4 disrupts this signaling axis, leading to the neuronal proliferative and migratory abnormalities characteristic of VMS [2,4,7]. Previous studies using global *Dchs1* knockout (*Dchs1^−/−^*) mice recapitulated several hallmark VMS phenotypes. Even though these mice are neonatal lethal, phenotypes observed at birth included craniofacial malformations and skeletal defects [6], as well as abnormal brain architectures [2]. These data support the *Dchs1^−/−^* mouse as a potential in vivo model of this human disease.

While the extracellular domain (ECD) of DCHS1 mediates heterophilic interaction with FAT4 [8], the function of its intracellular domain (ICD) remains poorly understood. The proteolytic processing of DCHS1 has been observed in multiple species and tissues [9,10], suggesting that cleaved fragments may participate in downstream signaling. Previous studies have also shown that upon DCHS1-FAT4 interaction, the complex is endocytosed by FAT4-expressing cells, which activate downstream PCP pathways [11]. This trans-endocytosis is mediated by the ICD of FAT4 [12]. However, the full physiological relevance and developmental role of the DCHS1 ICD have not been defined. Contradicting in vitro and in vivo studies have shown that altering the DCHS1 ICD in transfected human embryonic kidney cells does not affect DCHS1-FAT4 interactions [12] whereas alterations of the DCHS1 ICD in *Drosophilia* resulted in observed disruption of PCP [10]. To determine whether the function of the DCHS1 ICD was conserved in mammals, we generated mice in which the intracellular domain of DCHS1 was replaced with a V5 epitope tag (*Dchs1*^Δ*ICD-V5*^). We hypothesized that the deletion of the ICD would phenocopy the global *Dchs1^−/−^* knockout and reproduce many of the features of VMS, thereby revealing an essential role for the ICD in mediating Hippo pathway activity and tissue morphogenesis. Using structural, histological, and molecular analyses, we show that *Dchs1*^Δ*ICD-V5/*Δ*ICD-V5*^ homozygous mice are viable into adulthood, but exhibit craniofacial, skeletal, and neuronal defects comparable to those phenotypes observed in *Dchs1^−/−^* mice and VMS patients. These findings identify the DCHS1 intracellular domain as a critical effector of DCHS1–FAT4 signaling and establish its necessity for proper craniofacial and neural development.

## 2. Materials and Methods

### 2.1. Generation of Mouse Models

Generation and genotyping of *Dchs1* knockout mice (*Dchs1^−/−^*) were previously described [13]. The mouse strain C57BL/6J-*Dchs1^em1(HA)^* (termed *Dchs1^HA^*) was constructed and genotyped as previously described [9]. The mouse strain C57BL/6J-*Dchs1*^*em3(*Δ*R2955-I3291-V5)*^ (termed *Dchs1*^Δ*ICD-V5*^) was constructed by the Transgenomic Genome Editing Core at the Medical University of South Carolina and described as follows and in Figure S1A. To delete the intracellular domain (ICD) of *Dchs1* and introduce an in-frame C-terminal 2 × V5 tag (Figure 1F), two sgRNAs targeting the *Dchs1* locus were used (5′-CAGAGCTGCGCATCTAGCTG-3′ and 5′-TGTCTGTGACATCGGGCCAG-3′). A single-stranded DNA (ssDNA) repair template was included to support homology-directed repair (HDR) and to replace the excised ICD sequence with the 2 × V5 tag (Figure S1A). The ssDNA repair template sequence was TGGTCCTGGGCCTTGTTCGGGCGCGAAGCCGCAAGGCTGAGGCAGCtCCaGGAAGCGGGAAGCCTATCCCAAACCCCCTGTTGGGTCTCGACAGTACAGGCAGCGGCAAACCTATTCCAAATCCTCTGCTTGGTCTTGATAGCACTTgaCTGTGGCCCTAACTGGGCCCCGACCTGGGACACGTCCAGTGTCCCCAAAG. For CRISPR/Cas9 genome editing, sgRNAs, ssDNA repair template, and recombinant Cas9 protein (QB3 MacroLab, UC Berkely) were co-electroporated into C57BL/6J zygotes (The Jackson Laboratory, Bar Harbor, ME, USA; Stock #009086;) as previously described [9]. Electroporated embryos were cultured overnight at 37 °C in EmbryoMax Advanced KSOM (Merck & Co., Inc., Rahway, NJ, USA) to the two-cell stage, then transferred into pseudo-pregnant CD1 recipient females. Putative F0 founders were screened using tail biopsy genotyping. Candidate knock-in alleles were identified using PCR spanning the edited region of the *Dchs1* locus using primers 5′-CGATGGTCTGGTGCTCTACT-3′ and 5′-GACAGCAGGTGGGTTTCATC-3′. PCR products were analyzed using Oxford Nanopore long-read sequencing (Plasmidsaurus) to confirm correct editing and in-frame insertion of the tandem 2 × V5 tag downstream of the DCHS1 transmembrane domain (Figure S1B). Heterozygous mice with the V5-tagged mutated form of *Dchs1* (termed *Dchs1*^Δ*ICD-V5*^) were intercrossed. Pups, regardless of sex, from the resultant litters had their heads and brains excised for micro-computed tomography, Western blot analyses, and histological analysis. All functional analyses were performed at P0 due to the neonatal lethality of *Dchs1^−/−^* mice and to mitigate gestational differences associated with embryonic samples since structural and cellular phenotypes are robustly present at P0 allowing consistent assessment. Sample sizes (*n*) for each experiment are indicated in the corresponding methods and figure legends. All *n* values refer to biological replicates unless otherwise stated. All animal experiments were performed in accordance with IACUC procedures at Medical University of South Carolina under approved protocol number IACUC-2020–00956 (approval date: 27 January 2025). There are no human subjects involved in the study; thus, no relevant IRB approvals are needed.

### 2.2. Micro-Computed Tomography

Heads were excised from P0 litters. Mice were euthanized by decapitation. Heads were fixed in 4% paraformaldehyde for 4 h at room temperature and then washed in 1X PBS. Fixed mouse heads were then scanned with a SCANCO µCT 40 device (SCANCO Medical AG, Southeastern, PA, USA) at an isotropic voxel size of 8 µm. The following scanning parameters were used for acquiring bone morphometry: tube voltage, E = 55 kVp; tube current, I = 72 µA; and integration time, 200 ms. The resulting µCT datasets were imported into the Imaris software (Bitplane, Belfast, Northern Ireland, UK; version 10.1) for 3D reconstruction of skeletal structures using the “Surface” function. All µCT reconstructions were generated and visualized using identical scaling parameters.

A sample size of *n* = 3 of *Dchs1^−/−^*, *Dchs1*^Δ*ICD-V5/*Δ*ICD-V5*^, and control littermates was used for analysis and comparisons were made via a one-way ANOVA with significance *p* < 0.05. The width of sagittal suture of mouse skull was defined as the distance between medial margins of parietal bones [14]. Landmark placement and sagittal suture width measurements were performed on the 3D reconstructed mouse head using the “Measurement Points” function in Imaris. For each sample, measurements were performed blind to genotype, sagittal suture width was measured three times, and the average value was used for subsequent analysis.

### 2.3. Western Blot Analysis

Brains were excised from embryonic day 13.5 (E13.5), E18.5, and postnatal day 0 (P0) litters. For all timepoints (E13.5–P0), the mice were sacrificed via decapitation. After euthanasia, the brains were excised, digested in radioimmunoprecipitation assay (RIPA) buffer, minced with spring scissors, sonicated, and centrifuged (10 min at 17,000× *g* at 4 °C). Prior to Western blot analyses, protein concentration was determined by Bradford (Thermo Scientific, Waltham, MA, USA; #23236) and all samples were loaded equally (20 ug). Densitometric analyses were performed via Adobe Photoshop 2023 (Adobe Inc., San Jose, CA, USA) and normalized to total protein staining as determined by PonceauS (Thermo Scientific #161470250) to account for minor loading and transfer variability. For DCHS1-HA brain cleavage studies the following sample sizes were used: *n* = 6 for E13.5, *n* = 5 for E18.5, and *n* = 5 for P0. Comparisons between timepoints were analyzed via one-way ANOVA with significance *p* < 0.05. For *Dchs1*^Δ*ICD-V5/*Δ*ICD-V5*^ brain cleavage studies a sample size of *n* = 7 was used and comparison to P0 DCHS1-HA cleavage was made via an unpaired Student’s *t*-test. For detection of Hippo pathway mediators, Yap1 and phosphor-Yap1 a sample size of *n* = 3 was used. Protein lysates were probed using Western blotting and blinded to genotype as we have previously reported [9]. Membranes were incubated with primary antibodies against HA (Invitrogen, Waltham, MA, USA; #MA5-27915), V5 (Cell Signaling Technology, Danvers, MA, USA; #13202T), Yap1 (phospho-S127) (Abcam, Cambridge, UK; #ab76252), or Yap1 (Novus Biologicals, Centennial, CO, USA; #NB110-58358SS) at 1:1000 dilution, followed by goat anti-rabbit IgG-HRP (Sigma-Aldrich, St. Louis, MO, USA; #A8275). Bands were detected using SuperSignal West Femto Substrate (Pierce/Thermo Fisher Scientific, Waltham, MA, USA; #34096)

### 2.4. Alizarin Red/Alcian Blue Staining

Upper torsos including throat and head were excised from P0 litters preceded by decapitation. After excision, tissues were fixed in 4% paraformaldehyde for 4 h at room temperature, dehydrated and processed through graded alcohols, toluene, paraffinized, embedded in paraffin, and sectioned onto charged slides. For staining, slides were deparaffinized and rehydrated in xylenes and absolute alcohol. The exposed tissue was rinsed in distilled water and stained with an Alcian Blue solution made from Alcian Blue 8GX (Sigma-Aldrich, #A5268-25G) and 3% acetic acid (Sigma-Aldrich, A-6283) for 30 min at room temperature, washed in tap water, rinsed in distilled water and stained with an Alizarin Red solution made from Alizarin Red S (Sigma-Aldrich, A5533-25G) and distilled water for 2 min. The slides were dipped in acetone, acetone–xylene solution, and xylenes before being cover slipped with Cytoseal XYL (Epredia, Portsmouth, NH, USA; #8312-4). A sample size of *n* = 3 of *Dchs1^−/−^*, *Dchs1*^Δ*ICD-V5/*Δ*ICD-V5*^, and control littermates was used for analysis. Alizarin Red staining quantifications were measured via Adobe Photoshop 2023 (Adobe Inc., San Jose, CA, USA) and averaged per mouse between 3 separately stained slides (to account for potential staining differences) and fold change was determined as compared to control measurements. Results were analyzed via one-way ANOVA with significance *p* < 0.05. Circularity analysis was performed using ImageJ, version 1.53 (National Institutes of Health, Bethesda, MD, USA). Serial 10 μm tracheal sections starting below the epiglottis were measured until the point of decapitation (minimum 12 sections per mouse). Digital images were imported into ImageJ and calibrated for scale using the image metadata. Images were converted to 8-bit grayscale when necessary to improve contrast. The structure of interest was identified as the luminal surface of the tracheal epithelium. The luminal boundary was manually outlined using the freehand selection (drawing) tool to create regions of interest (ROIs). All measurements were performed using identical ImageJ settings for all images. Results analyzed were via two-way ANOVA with significance *p* < 0.05.

### 2.5. Immunohistochemistry

Following decapitation, heads of P0 mice were excised, fixed, embedded, and sectioned as described above. Slides were stained as previously described [9] and blinded to genotype. The following primary antibodies and reagents were used for immunostaining: rabbit α-HA antibody (Cell Signaling #3724) at a dilution of 1:250, mouse α-GFAP antibody (Santa Cruz Biotechnology #sc-33673) at 1:100, rabbit α-V5 antibody (Cell Signaling #13202T) at 1:100, and rabbit α-Ki67 antibody (Abcam # ab15580) at 1:100. Additionally, wheat germ agglutinin (WGA) pre-conjugated to Alexa Fluor 488 (Invitrogen #W11261) and NeuroTrace Nissl stain pre-conjugated to 640/660 (Invitrogen # N21483) were used at a 1:250 dilution. A sample size of *n* = 4 of *Dchs1^−/−^* and *Dchs1*^Δ*ICD-V5/*Δ*ICD-V5*^ mice was used for all analyses. Proliferation was assessed using Ki67 immunostaining quantified using Adobe Photoshop 2023 (Adobe Inc., San Jose, CA, USA). Digital images were imported into Photoshop and individual fluorescence channels were separated. Background staining in images was reduced using uniform background subtraction and intensity thresholding applied equally across all samples. Three regions of interest (ROIs) were identified per sample along the subventricular zone with a background measurement (quantified using the same size ROI in the deep ventricular tissue) subtracted from each measurement. The 3 ROI measurements were averaged to account for staining differences throughout the tissue. The averaged value referred to one biological replicate. Results were analyzed via unpaired *t*-test with significance defined as *p* < 0.05. Co-localization between Ki67 and NeuroTrace was quantified using ImageJ, version 1.53 (National Institutes of Health, Bethesda, MD, USA). The Manders’ overlap coefficient was calculated with consistent thresholding parameters applied across all samples. Digital images were imported into ImageJ and individual fluorescence channels were separated and converted to 16-bit images. Co-localization between individual channels was analyzed with values ranging from 0 to 1. All analyses were performed using identical ImageJ settings and blinded to genotype or treatment condition. Results were analyzed via unpaired *t*-test with significance defined as *p* < 0.05.

## 3. Results

### 3.1. DCHS1 Undergoes Proteolytic Processing During Brain Development

Mice with a targeted insertion of the Hemagglutinin (HA) tag in the *Dchs1* locus (*Dchs1^HA^*) were used to assess the endogenous expression of DCHS1 in the brain throughout fetal (embryonic day 13.5, E13.5, and E18.5) as well as neonatal (postnatal day 0) development using Western blot analyses. Whole-brain lysate revealed a high molecular weight band (>250 kDa) as well as a smaller band around 50 kDa (Figure 1B–D). Quantification of the full-length DCHS1, short-form (SF), and the ratio of short-form:full-length DCHS1-HA revealed consistent expression levels between embryonic (E13.5) and fetal (E18.5) timepoints with the short form of DCHS1 showing more intense banding during these timepoints in the brain. The presence of the full-length and short-form DCHS1 bands decreases by P0 resulting in a slight, but significant increase in the ratio of short-form:full-length DCHS1 expression (Figure 1E). Mice with a targeted deletion of the intracellular domain (ICD) of DCHS1 and replacement with a V5 tag (*Dchs1*^Δ*ICD-V5/+*^) were used to assess the endogenous expression of mutant DCHS1-dICD-V5 protein in the neonatal (P0) mouse brain. Whole-brain lysate revealed a molecular weight band (>250 kDa) as well as a smaller band around 15 kDa (Figure 1G). Much like in the context of the full-length DCHS1 protein, the short ~15kDa form appears to be the dominant form. These data allow us to roughly map a potential cleavage site on the extracellular surface of the DCHS1 protein near the level of the membrane (Figure 1H).

### 3.2. Deletion of the DCHS1 Intracellular Domain Recapitulates Craniofacial Abnormalities

**Figure 2:**
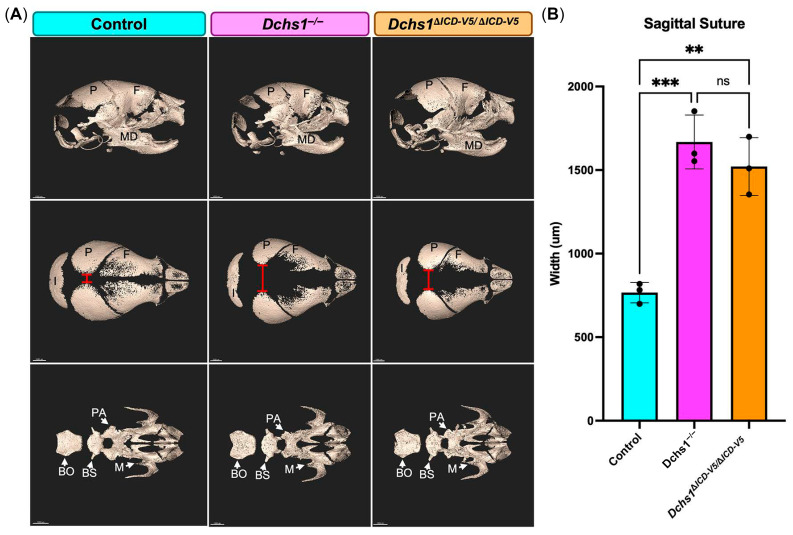
Micro-computed tomography of mutant Dchs1 mouse skulls. (**A**) Representative micro-computed tomography (*μ*CT) scans displaying the sagittal (top row) and cranial–caudal (middle row) views of P0 control (blue), global Dchs1 knockout (*Dchs1^−/−^*, pink), and homozygous ICD mutant *Dchs1*^Δ*ICD-V*5/Δ*ICD-V*5^ (orange) skulls. The lower row displays segmentation of the cranial base and upper jaw. (**B**) Quantification of the gap between sagittal sutures (red line in (**A**)) of *n* = 3 biological replicates per genotype (individual P0 skull). P, parietal; F, frontal; MD, mandible; I, interparietal; PA, palatine; BO, basioccipital; BS, basisphenoid; M, maxilla. One-way ANOVA: ns = nonsignificant; ** *p* < 0.01; *** *p* < 0.001. Scale bar 1000 μm.

*Dchs1* global knockout (*Dchs1^−/−^*) mice exhibit neonatal lethality and thus all studies on *Dchs1^−/−^* and mutant *Dchs1* mice lacking the intracellular domain (*Dchs1*^Δ*ICD-V5/*Δ*ICD-V5*^) were performed immediately after birth on postnatal day 0 (P0) when cellular and structural phenotypes were additionally consistently robust. Skulls from control, *Dchs1^−/−^*, and *Dchs1*^Δ*ICD-V5/*Δ*ICD-V5*^ mice were used to assess gross skeletal differences. Sagittal views from 3D reconstructed images revealed shortened nasal bones and mandibles of the *Dchs1^−/−^* and *Dchs1*^Δ*ICD-V5/*Δ*ICD-V5*^ mice compared to their control littermates (Figure 2A, top row). Cranial to caudal views revealed enlarged anterior and posterior fontanelles in the *Dchs1^−/−^* and *Dchs1*^Δ*ICD-V5/*Δ*ICD-V5*^ mice compared to control littermates (Figure 2A, middle row). Segmentation of the cranial base and upper jaw revealed smaller palatine and maxillary bones in the *Dchs1^−/−^* and *Dchs1*^Δ*ICD-V5/*Δ*ICD-V5*^ mice compared to control littermates (Figure 2A, bottom row). Although mutant skulls may appear smaller in the representative images, complete study cohorts did not indicate a reduction in overall skull size, suggesting that the phenotype primarily reflects altered craniofacial proportions rather than global scaling. Quantification of the shortest distance along the sagittal suture revealed an increased distance in the *Dchs1^−/−^* and *Dchs1*^Δ*ICD-V5/*Δ*ICD-V5*^ mice compared to control littermates, but no statistical differences in width were observed between the complete *Dchs1^−/−^* and *Dchs1*^Δ*ICD-V5/*Δ*ICD-V5*^ mice (Figure 2B).

### 3.3. Loss of DCHS1 ICD Disrupts Airway Cartilage Structure

**Figure 3:**
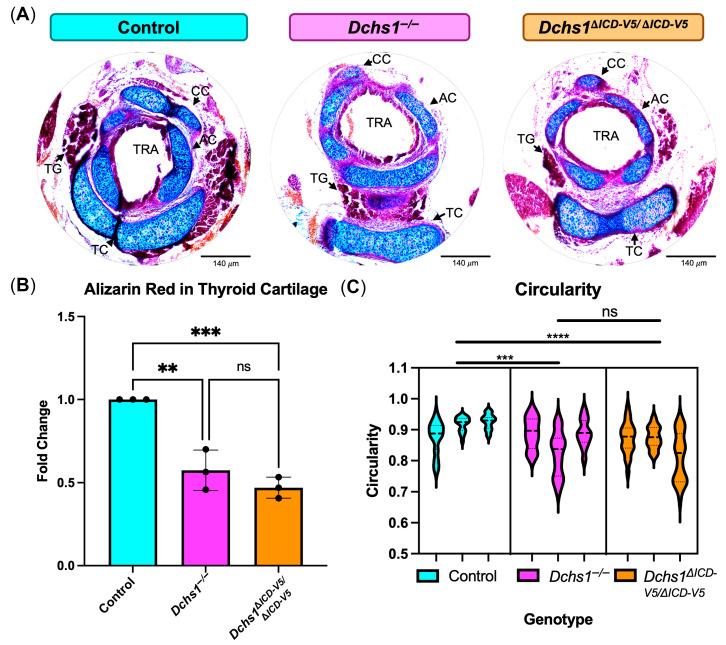
Alizarin Red/Alcian Blue staining of mutant Dchs1 tracheas. (**A**) Representative Alizarin Red/Alcian Blue staining of P0 control (blue), global Dchs1 knockout (*Dchs1^−/−^*, pink), and homozygous mutant *Dchs1*^Δ*ICD-V5/*Δ*ICD-V5*^ (orange) tracheas. (**B**) Quantification of Alizarin Red staining in thyroid cartilage of *n* = 3 biological replicates per genotype (individual P0 animal). (**C**) Quantification of circularity of the trachea as determined through serial 10 um sections starting below the epiglottis. Labeled abbreviations: CC, cricoid cartilage; TRA, trachea; AC, arytenoid cartilage; TG, thyroid gland; TC, thyroid cartilage. For (**B**): one-way ANOVA. For (**C**): two-way ANOVA. ns = non-significant; ** *p* < 0.01; *** *p* < 0.001; **** *p* < 0.0001. Scale bar 140 μm.

Serial 10 μm tracheal sections starting below the epiglottis from control, *Dchs1^−/−^*, and homozygous mutant *Dchs1*^Δ*ICD-V5/*Δ*ICD-V5*^ mice were stained with Alizarin Red/Alcian Blue to assess osteo-/chondrogenic and gross morphological changes. The location of cricoid cartilage (CC) posterior to the trachea varied from midline in control mice to more lateral positions in *Dchs1^−/−^* and *Dchs1*^Δ*ICD-V5/*Δ*ICD-V5*^ mice (Figure 3A). A lack of uniform Alcian Blue and Alizarin Red staining was observed throughout the pharynx cartilage in all mice with higher density staining observed around the periphery and decreased staining in the center of the observed cartilage. *Dchs1*^Δ*ICD-V5/*Δ*ICD-V5*^ mice, specifically, appeared to have less Alcian Blue staining throughout all pieces of pharynx cartilage (Figure 3A). Quantification of Alizarin Red revealed decreased Alizarin Red staining in both *Dchs1^−/−^* and *Dchs1*^Δ*ICD-V5/*Δ*ICD-V5*^ mice compared to controls (Figure 3B). Quantification of the circularity of the trachea was performed on all serial sections starting below the epiglottis to where the pup was decapitated. Two-way ANOVA analysis revealed decreased circularity in *Dchs1^−/−^* and *Dchs1*^Δ*ICD-V5/*Δ*ICD-V5*^ mice with more severe decreases in circularity seen in *Dchs1^−/−^* mice (Figure 3C).

### 3.4. DCHS1 Intracellular Domain Is Required for Polarized Localization in the Developing Brain

**Figure 4:**
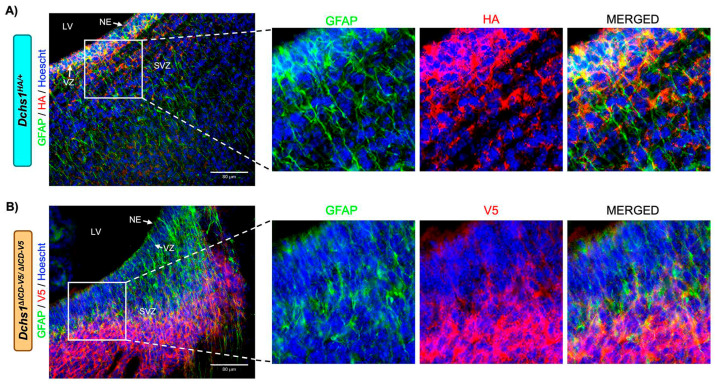
DCHS1 protein localization in neonatal mouse brain. (**A**,**B**) Coronal view of P0 brain surrounding the lateral ventricle of *Dchs1^HA/+^* (**A**) and *Dchs1*^Δ*ICD-V5/*Δ*ICD-V5*^ (**B**) mice stained for HA (red), V5 (red), Hoescht (nuclei blue), and GFAP (green). Notice the lack of expression in the neuroepithelium and an overall loss of polarity in the ICD mutant mice (enlarged boxed regions). LV, lateral ventricle; NE, neuroepithelium; VZ, ventricular zone; SVZ, subventricular zone. Scale bar 80 μm.

To analyze the brains of the *Dchs1* mutant animals, immunohistochemistry was performed on tissues surrounding the lateral ventricle of *Dchs1^HA/+^* and *Dchs1*^Δ*ICD-V5/*Δ*ICD-V5*^ mice (Figure 4). This enabled us to localize DCHS1 control and mutant proteins in vivo. Expression of the control DCHS1-HA protein was observed throughout the subventricular zone (SVZ), with increased intensity observed in the ventricular zone (VZ) and along the neuroepithelium (NE). DCHS1 was also observed to be organized in clustered staining that was perpendicular to astrocyte-specific marker, GFAP, in the subventricular zone (Figure 4A). In contrast, V5 staining, a surrogate for the expression of the mutant DCHS1-dICD-V5 protein, was observed uniformly throughout the subventricular zone with scant expression seen in the ventricular zone and along the neuroepithelium. The *Dchs1*^Δ*ICD-V5/*Δ*ICD-V5*^ mice appeared to lose polarized expression of the DCHS1 protein within the SVZ when the ICD was lost (Figure 4).

### 3.5. Loss of DCHS1 ICD Is Associated with Altered Hippo Pathway Activation and Increased Proliferation

**Figure 5:**
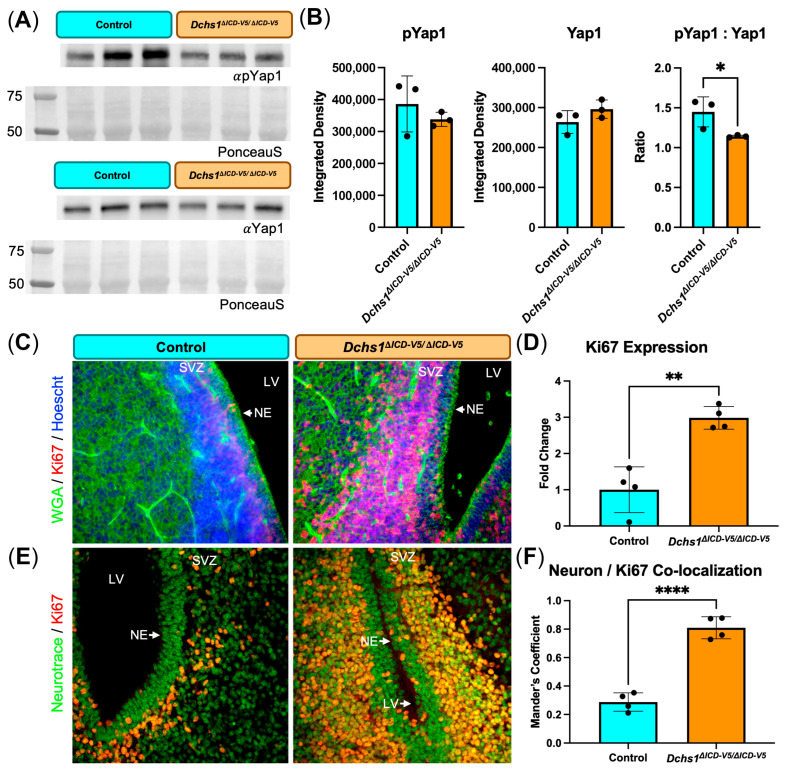
Altered Yap and proliferation in *Dchs1*^Δ*ICD-V5/*Δ*ICD-V5*^ mouse brain. (**A**) Whole-brain lysates of *n* = 3 P0 control (blue) and *Dchs1*^Δ*ICD-V5/*Δ*ICD-V5*^ (orange) pups probed for phosphorylated Yap1 (pYap1) and unphosphorylated Yap1 (Yap1) and Ponceau-S total protein staining. (**B**) Quantification of the integrated density of pYap1, Yap1, and the ratio of pYap1:Yap1 from A showing a statistically significant change in Yap levels. (**C**,**D**) Representative IHC for WGA and Ki67 and quantification of Ki67 staining of *n* = 4 control (blue) and *Dchs1*^Δ*ICD-V5/*Δ*ICD-V5*^ (orange) P0 brains showing robust increased proliferation within the subventricular zone (SVZ) of the mutant mice, which was predominantly localized to neurons using NeuroTrace (**E**,**F**). LV, lateral ventricle; NE, neuroepithelium; SVZ, subventricular zone. Scale bar 80 μm. Unpaired Student’s *t*-test: * *p* < 0.05; ** *p* < 0.01; **** *p* < 0.0001.

Due to previous reports demonstrating Dachsous–Fat as an upstream regulator of the Hippo pathway in *Drosophila* and other mammals [15], as well as the cellular and structural phenotypes observed in the *Dchs1* mutant animals, we analyzed whole-brain lysates for molecular changes in the Hippo pathway mediator Yap1 (Figure 5). Whole-brain P0 tissue lysate from control and *Dchs1*^Δ*ICD-V5/*Δ*ICD-V5*^ mice were probed via Western blot analyses for mediators of the Hippo pathway, phosphorylated Yap1 (pYap1) and unphosphorylated Yap1 (Figure 5A). Quantification of the ratio of pYap1:Yap1 revealed slight, but decreased ratios in *Dchs1*^Δ*ICD-V5/*Δ*ICD-V5*^ brain tissue (Figure 5B). Immunohistochemical staining of brain tissue surrounding the lateral ventricle in P0 control and *Dchs1*^Δ*ICD-V5/*Δ*ICD-V5*^ mice was used to analyze the proliferation marker Ki67 and membranous marker WGA (Figure 5C). Ki67 staining was observed in the subventricular zone in both conditions; however in the control mouse Ki67 staining was restricted to a relatively thin distribution directly next to the ventricular zone compared to the *Dchs1*^Δ*ICD-V5/*Δ*ICD-V5*^ mouse in which Ki67 was observed throughout the entirety of the subventricular zone. Quantification of the Ki67 proliferation index revealed increased proliferation in *Dchs1*^Δ*ICD-V5/*Δ*ICD-V5*^ brain tissue (Figure 5D). Immunohistochemical staining of brain tissue surrounding the lateral ventricle in P0 control and *Dchs1*^Δ*ICD-V5/*Δ*ICD-V5*^ mice was additionally used to analyze Ki67 staining in comparison to the neuron marker NeuroTrace Nissl stain (Figure 5E). The Manders’ coefficient between Ki67 and NeuroTrace was increased in *Dchs1*^Δ*ICD-V5/*Δ*ICD-V5*^ brain tissue staining suggesting increased neuronal proliferation (Figure 5F).

## 4. Discussion

*DCHS1* and *FAT4* encode large, atypical cadherin-family cell-surface proteins that function as a conserved receptor–ligand pair to coordinate tissue morphogenesis [8]. This pathway is best known for roles in planar cell polarity (PCP) and growth control via Hippo signaling, in which intercellular DCHS1–FAT4 interactions convert cell–cell contact information into polarized organization, regulated proliferation, and patterned differentiation [1,6,11,12,16,17,18,19]. In humans, the disruption of this axis is strongly linked to congenital malformation syndromes and neurodevelopmental disease: biallelic pathogenic variants in either gene cause Van Maldergem Syndrome (VMS), characterized by craniofacial and skeletal abnormalities, intellectual disability, and brain malformations including periventricular neuronal heterotopia [2,5,20]. *FAT4* variants are also associated with the clinically overlapping Hennekam spectrum [20] and DCHS1 mutations have been found in patients with autosomal dominant mitral valve prolapse (MVP) [21]. Despite this strong genetic foundation, the mechanistic basis by which DCHS1–FAT4 signaling is executed in mammalian tissues remains incomplete.

An unresolved issue is how the DCHS1 intracellular domain (ICD) contributes to DCHS1–FAT4 function in vivo. While the DCHS1 ectodomain mediates FAT4 binding and has been implicated in PCP and Hippo pathway regulation [1,6,11,12,16,17,18,19], the physiological role of the ICD has remained unclear, with conflicting conclusions from in vitro systems [12] and invertebrate models [10] that may not capture mammalian tissue architecture, heterotypic cell neighborhoods, or the spatial logic of polarity establishment. To directly address this gap, we generated and characterized a mouse model in which the DCHS1 ICD is deleted and replaced with an epitope tag, preserving the extracellular and transmembrane regions while functionally uncoupling extracellular recognition from intracellular signaling capacity. This strategy allowed us to define ICD-dependent functions in vivo and to test whether the ICD is required for DCHS1 polarization, Hippo pathway output, and coordinated craniofacial and neural morphogenesis.

Our findings demonstrate that targeted ICD deletion disrupts cell polarization in the developing brain with associated changes in Hippo pathway activity, and that these changes are accompanied by craniofacial, skeletal, and neural abnormalities that closely resemble those seen in global *Dchs1*-null mice [6,13] and in VMS [2,4]. Mechanistically, we propose that a key function of the DCHS1 ICD is to act as a cytoskeletal tether, linking membrane-localized DCHS1/FAT4 complexes to intracellular scaffolds such as septin networks and tubulin-based microtubules as has previously been shown [22,23]. Through this mechanism, this complex could enable the transmission of heterotypic cell–cell contact spatial cues. In this context, the ICD emerges as an essential determinant of mammalian DCHS1 signaling by functioning in the organization of receptor complexes within the plane of the tissue, maintaining asymmetric localization, and integrating adhesion with cytoskeleton-dependent remodeling that underlies PCP-like cues and contact-dependent growth. These in vivo results help reconcile discrepancies in prior work by showing that mammalian developmental phenotypes and tissue-level organization are sensitive to ICD-dependent coupling between membrane complexes and the cytoskeleton.

A second major implication of our work is that *DCHS1* biology in mammals appears to be shaped by regulated proteolytic processing near the transmembrane domain. Consistent with prior reports of DCHS1 proteolysis [9,10], we detected both full-length protein and a prominent ~50 kDa short form across embryonic, fetal, and early postnatal stages in *Dchs1^HA/+^* mice, with the short form predominating throughout development. Because the HA epitope maps to the extreme C-terminus of the cytoplasmic tail, this band is most consistent with a stable C-terminal fragment (CTF) that retains the ICD and likely the transmembrane helix. The observed mobility relative to the predicted ~37 kDa ICD suggests inclusion of the transmembrane region and a small portion of the proximal extracellular ectodomain, potentially extending into the first two cadherin repeats (approximately amino acids ~2800–3298). Importantly, this region lacks evidence for an internal translation start site or strong Kozak context that would support an independent ~50 kDa isoform, favoring proteolytic cleavage rather than alternative initiation. As a type I single-pass membrane protein, DCHS1 is well positioned to generate discrete CTFs via ectodomain shedding within an extracellular domain near the membrane, commonly mediated by metalloprotease sheddases such as ADAM10/ADAM17 [24]. In support of this mechanism, the sequence immediately N-terminal to the predicted transmembrane helix is low-complexity and enriched in small/polar residues, consistent with an accessible near-TM cleavage region. The abundance and developmental predominance of this CTF therefore suggest that regulated juxtamembrane cleavage may be a key mechanism for tuning DCHS1 surface abundance and downstream signaling during development.

Notably, cleavage was conserved in the ICD deletion allele, supporting the interpretation that DCHS1 processing is not simply a by-product of ICD-mediated signaling but rather reflects an intrinsic, regulated event governed by membrane-proximal extracellular sequence features. This conservation has two important implications. First, it argues that the developmental phenotypes in ICD-deleted animals do not arise from the loss of proteolysis per se, but from the loss of ICD-dependent coupling between extracellular events and intracellular responses. Second, it raises the possibility that following ectodomain cleavage, the remaining membrane-tethered DCHS1 fragment may have additional roles in development beyond serving as an inert degradation product. In other large type I membrane proteins, cleavage can repurpose the remaining fragment as a signaling scaffold, alter receptor complex stoichiometry, or create a substrate for additional processing steps [12,24,25]. For DCHS1, analogous processing could dynamically regulate how DCHS1–FAT4 complexes assemble, how long they persist at specific interfaces, and how effectively they engage downstream pathways in a tissue- and stage-dependent manner.

Our brain analyses support a framework in which the ICD is required to translate DCHS1–FAT4 interactions into polarized organization and growth control. In control tissue, DCHS1 shows organized, polarized enrichment along a neuroepithelial architecture, consistent with its established links to PCP and ventricular zone organization [2]. By contrast, ICD-deleted DCHS1 fails to concentrate appropriately and is diffusely distributed, indicating that the ICD is necessary for subcellular localization and the establishment or maintenance of polarity cues during neurodevelopment. This is conceptually aligned with *Drosophila*, where Dachsous–Fat signaling functions as a polarity system that converts intercellular differences into planar organization [10]. Our data suggest that mammalian DCHS1 likewise depends on its ICD to execute this polarity logic in vivo. Importantly, polarity disruption provides a mechanistic bridge to our Hippo pathway findings and to the proliferation phenotypes observed in mutant mice. We propose that the ICD-dependent organization of DCHS1 at cell–cell interfaces is required for the appropriate contact-dependent restraint of proliferation (i.e., effective contact inhibition), particularly within heterotypic cellular neighborhoods such as the ventricular/subventricular zones, where progenitors, radial glia scaffolds, and differentiating neuronal populations form dynamic, asymmetric contacts [2]. In this context, the loss of the intracellular domain (ICD) would be expected to weaken DCHS1–FAT4 signaling and thereby reduce Hippo-mediated growth control, promoting excess proliferation and aberrant differentiation within neurogenic niches as was seen in the presented study. This is an axis that could plausibly contribute to heterotopia-associated phenotypes as observed in VMS. That said, the Hippo pathway effect observed here appears modest (based on YAP1 readouts) and is unlikely, on its own, to account for the pronounced proliferative phenotype in the ICD mutant. This discrepancy suggests that additional, more dominant pathways are driving proliferation in this context. Additional studies examining upstream regulators, downstream transcriptional outputs, and the functional rescue of Hippo signaling and other proliferation pathways will be required to establish a direct mechanistic link.

Our skeletal and craniofacial phenotypes further underscore that the DCHS1 intracellular domain is indispensable for mammalian morphogenesis. ICD-deleted mice recapitulate hallmark features of global *Dchs1* deficiency [6] and mirror VMS-associated craniofacial findings [4], including craniofacial flattening, enlarged fontanelles, reduced maxillary/palatine structures, as well as defects in cartilage integrity and airway geometry. Strikingly, we observe complete neonatal lethality in *Dchs1^−/−^* animals, accompanied by a more severe tracheal phenotype than that seen in controls. Although the proximate cause of death has not been formally established, the severity of the airway defect is consistent with the possibility that neonatal lethality in the mice is driven, at least in part, by tracheal collapse, paralleling clinically significant airway compromise reported in VMS (manifesting as tracheomalacia) often requiring surgical intervention [4]. In contrast, *Dchs1*^Δ*ICD-V5/*Δ*ICD-V5*^ mice remain viable despite measurable tracheal abnormalities. This suggests that the preservation of the extracellular and transmembrane regions may provide partial structural or signaling function sufficient to maintain airway patency above a life-limiting threshold. This allelic series supports a graded model of DCHS1 function in which the ICD is required for full developmental output, but non-ICDs can retain limited activity that modulates phenotypic severity. Additionally, the difference in viability between *Dchs1^−^^/−^* and *Dchs1*^Δ*ICD-V5/*Δ*ICD-V5*^ mice supports a model in which DCHS1-mediated adhesion and intracellular signaling make distinct and non-equivalent contributions to developmental outcomes. Although *Dchs1*^Δ*ICD-V5/*Δ*ICD-V5*^ mice are viable, adult phenotypes were not assessed in this study, as our focus was on early developmental processes. Future studies will be required to determine whether ICD-dependent signaling contributes to postnatal tissue homeostasis or disease-relevant phenotypes.

The findings of this study are consistent with the impaired coordination of progenitor proliferation and development differentiation programs previously linked to DCHS1–FAT4 and possibly Hippo signaling [2,3,6,16,19,26]. Our results demonstrate that the ICD is a critical component of this axis, potentially reflecting bidirectional signaling as previously suggested by *Drosophila* studies [10]. Viewed together, our findings support an integrated model in which regulated near-transmembrane cleavage tunes DCHS1 availability and may generate functional membrane-tethered fragments. In parallel, the intracellular domain is required to translate extracellular, heterotypic cell–cell interactions into polarized organization and growth control that ultimately regulates craniofacial, skeletal, and neural development.

## 5. Limitations

Several limitations should be considered. First, cohort sizes for some analyses were necessarily modest due to the technical complexity of obtaining consistent anatomical planes and comparable alignments across samples and genotypes. While phenotypes were consistent across specimens and supported by multiple complementary approaches, future studies with larger cohorts and expanded quantitative morphometric and molecular analyses will be important to further validate and extend these findings. Second, although the ~50 kDa species is strongly supported to be a DCHS1 C-terminal fragment based on C-terminal detection and apparent size, its precise composition remains inferred; we have not mapped the exact cleavage site(s), defined the responsible protease(s), or established whether ectodomain shedding generates soluble extracellular products in vivo. Third, while the ΔICD allele preserves extracellular and transmembrane regions, replacing the native cytoplasmic tail with an epitope tag could influence trafficking or stability, and our current data do not resolve the degree to which phenotypes are cell-autonomous versus driven by disrupted heterotypic interactions within developing niches. Complementary alleles (motif-specific ICD mutants) and conditional/mosaic strategies may localize ICD requirements to specific lineages and interfaces. Finally, Hippo and polarity outputs were assessed with limited cell type and spatial resolution; future studies using reporters for progenitors and migrating neuron markers, quantitative polarity metrics, and live imaging will strengthen the causal links between ICD-dependent localization, contact inhibition, and Hippo pathway activity, and functional airway measurements will be needed to formally test whether tracheal collapse drives perinatal lethality in global *Dchs1*-null mice.

## 6. Conclusions

Collectively, our work establishes the DCHS1 intracellular domain as an essential determinant of mammalian morphogenesis that links extracellular DCHS1–FAT4 interactions to polarized tissue organization and suggests it has a role in Hippo-dependent growth control. By leveraging an ICD deletion allele that preserves the extracellular and transmembrane regions, we show that the loss of the ICD is sufficient to phenocopy the key craniofacial, skeletal, and neurodevelopmental abnormalities observed in global *Dchs1* deficiency and in VMS, while simultaneously disrupting DCHS1 polarization and Hippo pathway output in the developing brain. In parallel, the developmental predominance of a conserved ~50 kDa DCHS1 C-terminal fragment supports regulated juxtamembrane proteolysis as a core feature of DCHS1 biology and raises the possibility that cleavage products contribute additional, stage-specific functions during development. Together, these findings provide a unifying framework in which DCHS1 proteolytic processing and ICD-dependent signaling cooperate to regulate polarity, heterotypic cell–cell interactions, and contact inhibition, processes fundamental to coordinated growth, differentiation, and tissue architecture. These studies also define tractable mechanistic directions for future studies to map cleavage events, identify responsible proteases, and dissect cell-type-specific requirements for ICD function in neural and cardiac disease-relevant contexts.

## Data Availability

The original contributions presented in this study are included in the article/Supplementary Material. Further inquiries can be directed to the corresponding author.

## Supplementary Materials

The following supporting information can be downloaded at: https://www.mdpi.com/article/10.3390/cells15070587/s1, Figure S1: Dchs1^ΔICD-V5^ Mouse Strain Construction.
