# Supplementary material for: Loss of the DCHS1 Intracellular Domain Expands Neurogenic Proliferation and Generates Van Maldergem-like Neurodevelopmental Defects
Source: Cells. 2026 Mar 26;15(7):587. doi: 10.3390/cells15070587 (PMC13072099; doi:10.3390/cells15070587)
Supplement: Supplementary file 1 [file cells-15-00587-s001.zip › cells-4173347-supplementary.pdf]

Supplementary Figure Caption

Supplementary Figure S1. *Dchs1*$^{\Delta ICD\text{-}V5}$ Mouse Strain Construction

A) Schematic of the CRISPR/Cas9-mediated targeting strategy used to delete the endogenous *Dchs1* intracellular domain midway through the 21st exon and insert tandem V5 epitope tags, generating the p.Pro3247delins(V5x2) mutation at the C terminus of DCHS1 (resultant mouse strain termed *Dchs1*$^{\Delta ICD\text{-}V5}$). **B)** Genomic PCR analysis of putative F0 founders demonstrating correct modification of the *Dchs1* allele of the selected founder, as highlighted by the red box.

A

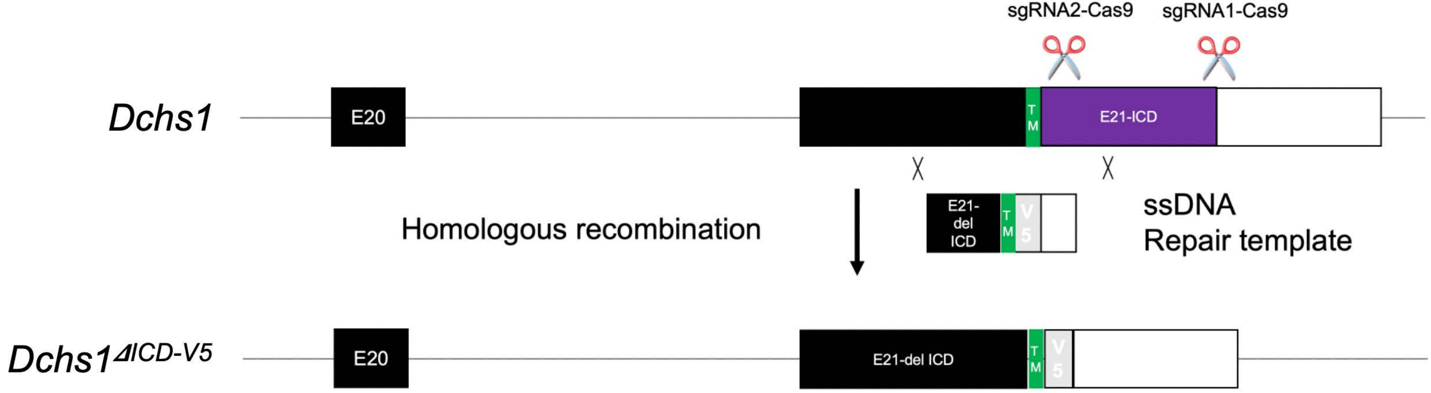
sgRNA2-Cas9
sgRNA1-Cas9
Dchs1
E20
T M
E21-ICD
Homologous recombination
E21- del ICD
V 5
ssDNA
Repair template
Dchs1ΔICD-V5
E21-del ICD

B

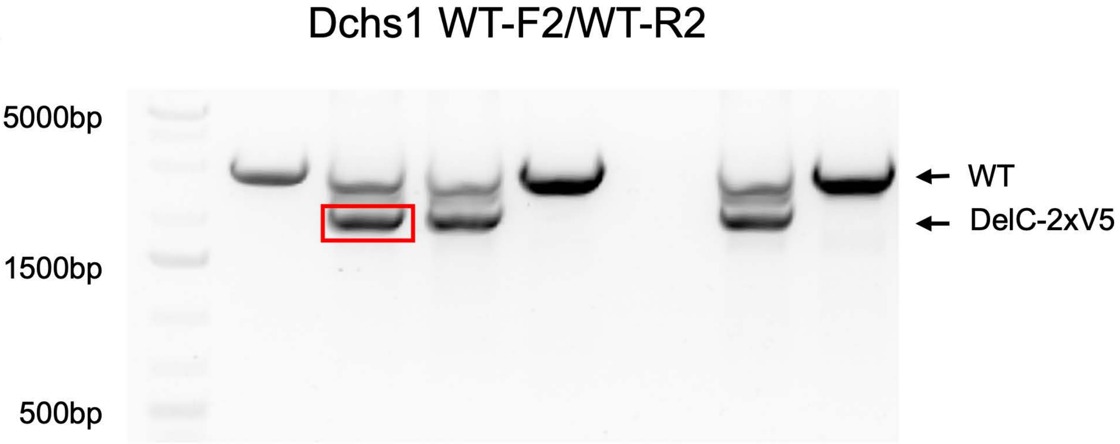
Dchs1 WT-F2/WT-R2
5000bp
1500bp
500bp
WT
DelC-2xV5

Figure S1
